# Metabolic modeling-based drug repurposing in Glioblastoma

**DOI:** 10.1038/s41598-022-14721-w

**Published:** 2022-07-01

**Authors:** Claudio Tomi-Andrino, Alina Pandele, Klaus Winzer, John King, Ruman Rahman, Dong-Hyun Kim

**Affiliations:** 1 Centre for Analytical Bioscience, Advanced Materials and Healthcare Technologies Division, School of Pharmacy, University of Nottingham, Nottingham, NG7 2RD UK; 2 Nottingham BBSRC/EPSRC Synthetic Biology Research Centre (SBRC), School of Life Sciences, BioDiscovery Institute, University of Nottingham, Nottingham, NG7 2RD UK; 3 Nottingham BBSRC/EPSRC Synthetic Biology Research Centre (SBRC), School of Mathematical Sciences, University of Nottingham, Nottingham, NG7 2RD UK; 4 Children’s Brain Tumour Research Centre, Biodiscovery Institute, School of Medicine, University of Nottingham, Nottingham, NG7 2RD UK

**Keywords:** Cancer, Computational biology and bioinformatics, Drug discovery

## Abstract

The manifestation of intra- and inter-tumor heterogeneity hinders the development of ubiquitous cancer treatments, thus requiring a tailored therapy for each cancer type. Specifically, the reprogramming of cellular metabolism has been identified as a source of potential drug targets. Drug discovery is a long and resource-demanding process aiming at identifying and testing compounds early in the drug development pipeline. While drug repurposing efforts (i.e., inspecting readily available approved drugs) can be supported by a mechanistic rationale, strategies to further reduce and prioritize the list of potential candidates are still needed to facilitate feasible studies. Although a variety of ‘*omics*’ data are widely gathered, a standard integration method with modeling approaches is lacking. For instance, flux balance analysis is a metabolic modeling technique that mainly relies on the stoichiometry of the metabolic network. However, exploring the network’s topology typically neglects biologically relevant information. Here we introduce *Transcriptomics-Informed Stoichiometric Modelling And Network analysis* (TISMAN) in a recombinant innovation manner, allowing identification and validation of genes as targets for drug repurposing using glioblastoma as an exemplar.

## Introduction

Cancer is the first or second cause of premature death in 70% of countries^1^. Tumors are characterized by multiple biological capabilities conferring cellular proliferation and metastasis (dissemination to other tissues and colonization), where metabolic reprogramming is crucial to fulfil biosynthetic requirements underlying these phenotypes^2^. The disparity of oxygen and nutrient availability within the surrounding tumor microenvironment promotes different metabolic states amongst cells, which show a continuum in gene expression within sub-clonal populations^3^. Consequently, metabolic dependencies and vulnerabilities are shaped by the cell lineage and the environment, rather than being universal^4^. Isocitrate dehydrogenase-1 wild-type Glioblastoma (GBM) is the most aggressive brain tumor in adults^5^, characterized by a high degree of invasiveness through brain parenchyma^6^. Different subtypes and transcriptional subpopulations may co-exist within the same tumor, where a quiescent subpopulation of GBM stem cells (GSC) can facilitate the development of radio- or chemo-resistance, leading to inevitable disease relapse^7–9^. Therefore, novel therapeutic approaches should aim to target all cell lineages^10^, block GSC differentiation^11^, or combine both strategies^9^. In particular, recent studies have shown the suitability of chemicals disrupting energy metabolism in GBM^12^ or blocking de novo pyrimidine synthesis in GSC^13^. Conversely, the application of immunotherapy and many drug compounds is hindered by the impermeability of the blood–brain–barrier^14^.

Phenotypic screening of compound libraries can help identify putative drug candidates, albeit being a costly process and requiring further target deconvolution steps^15^. Although gene essentiality is used as a proxy for potential drug targeting, its context-dependent and evolvable nature hinders its usage, for example: (i) genetic ablation affects both the catalytic activity (which may not be essential) and any other function (e.g. essential structural); (ii) incomplete knockdown or off-target effects; (iii) genetic divergence of cell lines from the original tumor; (iv) metabolic response of cells to non-physiological growth conditions^16–19^.

Whilst simulating ligand–target interactions (molecular docking) provides a shortlist of compounds to be tested, the scarcity of structural data limits its application^20^. In addition, this approach prevents the identification of compounds indirectly affecting metabolism, for example by altering the expression levels of multiple genes^21^. Therefore, information-rich computational strategies to generate manageable lists of drug candidates should be considered as a viable complement to the drug discovery phase. The set of metabolic fluxes (i.e., metabolic or transport reaction rates) can be either estimated via ^13^C metabolic flux analysis (^13^C-MFA) for the central carbon metabolism or modelled by applying flux balance analysis (FBA) to a genome-scale model (GSM).

A GSM consists of a stoichiometric matrix connecting metabolites (substrates and products) with reactions, where the latter are associated with genes following the gene-protein-reaction formalism^22^. FBA predicts a flux distribution upon the optimization for a biological task (normally the biomass formation), represented by a reaction in the GSM^23^. Whilst ^13^C-MFA mainly informs about the central carbon metabolism^4,24^, transcriptomics data can be applied to obtain cell-specific models from GSMs and can predict in silico gene essentiality^25–27^. Other strategies based on network analysis allow identification of highly connected reactions or reactions which exclusively consume/produce a metabolite (choke points) that if inhibited, would potentially disrupt metabolism^28^.

To this end, we developed *Transcriptomics-Informed Stoichiometric Modelling And Network analysis* (TISMAN), a recombinant innovation introducing the concept of “extended choke point” and exploiting biologically-rich information aiming at developing a workflow providing a stringent search of potential drug targets and compounds to be tested for GBM as an exemplar disease type. Briefly, published data were exploited to build condition specific GBM metabolic models, which were analyzed to identify and rank the reactions of interest according to different indicative criteria. Several chemical-gene interaction databases were used to identify and prioritize approved drugs or experimental compounds either directly targeting proteins or indirectly affecting the corresponding gene expression levels. Chemicals with a proven impact against other cancer types but which have not been tested for GBM, were prioritized for experimental validation using patient-derived GBM in vitro models.

## Materials and methods

**Figure 1:**
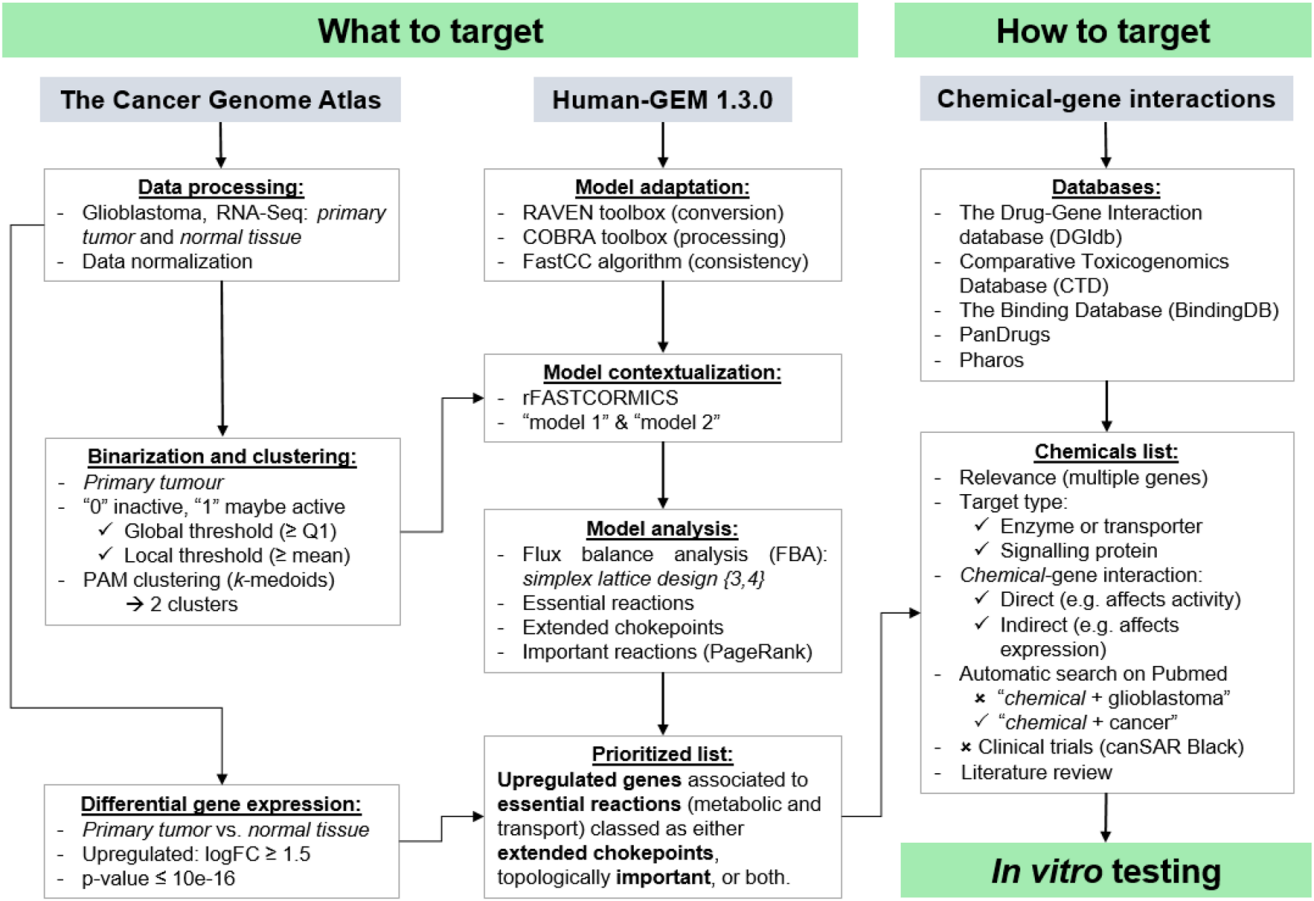
TISMAN (Transcriptomics-Informed Stoichiometric Modelling And Network analysis), the workflow developed in this study. It comprised three main parts (what to target, how to target and in vitro testing), where both MATLAB and R scripts were used.

The workflow developed in this study comprised three main parts (Fig. 1): (i) “what to target”: identification of either metabolic or transport (e.g., between cellular compartments) reactions of interest; (ii) “how to target”: identification of chemicals (readily available approved drugs or experimental compounds); (iii) in vitro validation of the selected compounds. Briefly, transcriptomics data from GBM tumors was used to: (a) contextualize a generic human GSM, yielding two GBM-specific models; (b) identify up-regulated genes. Next, the models were subjected to FBA to identify essential reactions, and the network topology was inspected to pinpoint certain features (choke points and centrality). Overall, the workflow aimed to identify proteins that if targeted, would potentially hinder cellular metabolism, impairing tumor metabolic viability and/or invasion. Chemicals targeting metabolism either directly (i.e., affecting the activity) or indirectly (i.e., affecting gene expression) were ranked, with five candidates prioritized for in vitro testing. Files and scripts for MATLAB and R can be found in Github (https://github.com/CTA-code/TISMAN).

### What to target

#### Transcriptomics data analysis

Data processing: RNA-Seq data for GBM and healthy counterpart astrocytic tissue (herein referred to as ‘primary tumor’ and ‘normal tissue’) was obtained from the Cancer Genome Atlas (TCGA) database (https://portal.gdc.cancer.gov/) and processed using the R package TCGAbiolinks^29^. This information was used for two independent steps, binarization and clustering. Expression data from *primary tumor* was binarized by considering two simultaneous thresholds: (i) global (≥ Q1) across all genes and samples, and (ii) local (≥ mean), which was gene-specific across samples. Genes were flagged as either *maybe active* (“1”) or *inactive* (“0”) if fulfilling both criteria or not, respectively, so that each sample was codified by a binary vector^25^. Next, R’s PAM function (Partitioning Around Medoids, or *k*-medoids) was used to cluster the data into two groups (optimal number of clusters according to the Silhouette method), yielding a representative binary vector (or medoid) for each cluster^30^. Focusing on upregulated genes in cancerous tissue compared to normal tissue is a widely used strategy to identify potential therapeutic genes^31^. Therefore, both *primary tumor* and *normal tissue* datasets were used to determine the differential gene expression: genes with logFC ≥ 1.5 and p-value ≤ 10^–16^ were deemed upregulated in the tumor^32^.

#### Stoichiometric modeling and network analysis

Model adaptation: the latest version of a human GSM (Human-GEM 1.3.0, comprising 13,417 reactions, 10,135 metabolites and 3,628 genes^33^) was converted and adapted to a COBRA toolbox paradigm by using the RAVEN toolbox^34^ and the COBRA toolbox^22^. Next, a consistent model was generated by FastCC prior to the contextualization step, so that all reactions were active (i.e., nonzero flux) for at least one feasible flux distribution^35^.

Model contextualization: the two medoids were used with rFASTCORMICS^27^ to build two cancer-specific models (COBRA toolbox v. 2, with IBM CPLEX 12.8 in MATLAB R2016b run in Windows 10 to ensure solver compatibility).

**Figure 2:**
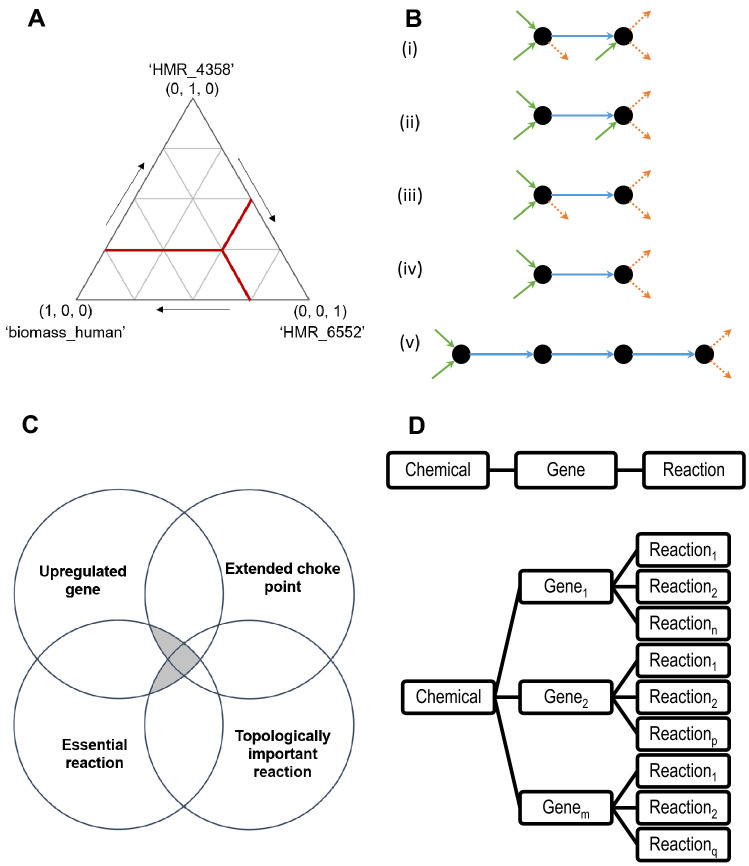
Strategies to identify gene targets for drug repurposing. (**A**) Simplex lattice design $\left\{\mathrm{3,4}\right\}$ for objective function weight values distribution. Each outer vertex represents a case where a single objective function is selected: maximization of the biomass yield (‘biomass_human’), maximization of the ATP yield (‘HMR_4358’), and maximization of 1-phosphatidyl-1D-myo-inositol-4-phosphate production (‘HMR_6552’). For illustrative purposes, the test comprising weight values (0.25, 0.25, 0.50) is labeled in red. (**B**) Examples of metabolites-reactions relationships (dots and arrows, respectively). The blue arrows represent the production of the metabolite on the right from the metabolite on the left. Inward green arrows refer to production reactions from metabolites not drawn, while outward orange dotted arrows represent consumption reactions. (i) Not a choke point, (ii) single choke point on the consumption side, (iii) single choke point on the production side, (iv) double choke point, and (v) extended choke point. (**C**) Venn diagram showing the subsets of reactions of interest. In all cases, reactions (metabolic or of transport) were essential and carried out by proteins related to upregulated genes, as well as identified as extended choke point and/or topologically important reactions. (**D**) (Top) Direct chemical–gene interaction, where a compound affects the activity of a single protein (e.g., an inhibitor). (**D**) (Bottom) Indirect chemical–gene interaction, where a single compound targets an element such as a signaling protein, that leads to changes in the expression levels of multiple genes, thus affecting several reactions in a pleiotropic manner.

Model analysis: an upper bound of 2 mmol/gDW-h was set for the oxygen uptake rate, as suggested for previous cancer models^36,37^. For the sake of modelling a variety of metabolic states, several objective functions were considered: maximization of the biomass yield (‘biomass_human’), maximization of the ATP yield (‘HMR_4358’), and maximization of 1-phosphatidyl-1D-myo-inositol-4-phosphate production (‘HMR_6552’), a lipid which induces curvature in the lipid membrane^38^. The latter reaction is catalyzed by synaptojanin 2 (*SYNJ2*) a polyphosphoinositide phosphatase related to GBM invasion^39^ and a mediator of metastasis in breast cancer^40^. Instead solving a multi-objective linear problem^41^, the weighted global criterion method was used to reformulate it as a single objective problem directly solvable by FBA^23,42^. Therefore, a simplex lattice design for FBA was designed by varying the weight of the three reactions, allowing to study the aforementioned three objective functions and mixtures amongst them in 15 single objective problems (Fig. 2A). In addition, an extra case for the minimization of the sum of fluxes was considered, yielding 16 final tests. Since certain optimal solutions were the same, the number of non-redundant solutions for each model was calculated. Reactions of interest fulfilled several criteria (Fig. 2C): related to an upregulated gene in the tumor; essential reaction; extended choke point and/or topologically important. Considering the full GSM and the flux through the biomass reaction when maximizing for it in FBA, a reaction was deemed essential for a test (same network, different objective function(s)) when shutting it down (no flux through allowed) caused a ≥ 5% reduction of said metabolic flux. Conversely, topological aspects were studied by exploring reduced metabolic networks. A condition-specific model was generated for each unique flux distribution produced by FBA, so that the stoichiometric matrix only comprised active elements (reactions and metabolites). Single choke points were identified as reactions associated exclusively with producing or consuming a metabolite. Double choke points were then defined as reactions associated exclusively with producing and consuming metabolites. To ensure a more stringent selection criterion, the concept of extended choke points was introduced, defined as double choke points surrounded by single choke points (consuming and producing) (Fig. 2B). Alternatively, topologically important reactions were identified by MATLAB’s in-built PageRank function^43^. This analysis was applied to a directed graph (i.e., considering the flux direction) derived from an adjacency matrix, where the centrality of the nodes (reactions) was determined. Directly calculating the adjacency matrix from the stoichiometric matrix would yield a metabolite-centric graph (metabolites as nodes)^44^. Therefore, an intermediate step converting the stoichiometric matrix into its conjugate transpose (reactions as nodes) was included prior to generating the adjacency matrix.

Prioritized list: reactions of interest for each medoids were identified by independently selecting those identified as extended choke points in at least half of the non-redundant optimal solutions (occurrence), and those within the top 10% of central reactions according to PageRank. Finally, results from both medoids were combined by summation of occurrence or centrality scores, respectively.

### How to target

#### Identification of relevant drugs and chemicals

Five databases were used, either programmatically or manually: *The Drug-Gene Interaction database* (DGIdb) Nov 2020, v4.2.0^45^; *Comparative Toxicogenomics Database* (CTD), Oct 2020^46^; *The Binding Database* (BindingDB), Nov 2020^47^; *PanDrugs*, version: 2018.11.7^48^; and *Pharos*, version 3.2.0^49^. To exploit published chemical–gene interactions, a gene-centric list of potential candidates was generated from the reactions of interest. Since a gene may participate in several reactions, occurrence and centrality scores for each gene were normalized and separately summated. Similarly, as a chemical may target (directly or indirectly) one or several genes (Fig. 2D), drug-centric lists were created, and a final relevance score was calculated as the sum of occurrences and centrality scores. Automatic searches on Pubmed were performed to identify which drugs have been tested for cancers (except for GBM), followed by a literature review and search for clinical trials in canSAR^50^ to finally select five compounds for in vitro testing.

### In vitro testing

#### Cell culture

**G**lioblastoma **IN**vasive margin (GIN28, GIN31) and **G**lioblastoma **C**ontrast-**E**nhanced core (GCE28, GCE31) cell lines have been previously derived at Queen’s Medical Centre, Nottingham, authenticated using short tandem repeat genotyping (Supplementary Table S1) and used at passages 21–29, 27–36, 15–23 and 28–37, respectively^51,52^. Cell lines were cultured using adherent T75 flasks (Corning) in Dulbecco’s Modified Eagle Medium (Gibco) supplemented with 10% FBS (Sigma-Aldrich) and incubated under humidified growth conditions at 37 °C and 5% CO_2_.

#### Metabolic viability assay

All cell lines were seeded in 96 well plates (Costar) at 5 × 10^3^ cells/well and incubated for 24 h prior to treatment. Cells were treated with afuresertib (Selleckchem), isorhamnetin (Merck), formononetin (Merck), pyrogallol (Merck) and taxifolin (Merck) in a 16-point dose response (n = 6) over 72 h, with concentrations ranging from 1 to 500 μM. Appropriate vehicle (0.1% DMSO) and positive cell death controls (10% DMSO) were used. Viability was assessed by adding 1:10 PrestoBlue (ThermoFisher) over 45 min and measuring the resulting fluorescence at 544/590 nm (excitation/emission) on a FLUOstar Omega microplate reader (BMG LABTECH). Fluorescence intensity was normalized against that of control wells to determine percentage viability of drug-treated cells.

#### Invasion assay

The ability of the compounds to inhibit invasion was investigated for all cell lines using a Transwell collagen barrier assay. 24 well ThinCert™ 8 µm cell culture inserts (Greiner) were coated with 10 µg of mouse collagen IV (Cultrex, Trevigen) and incubated at 37 °C overnight. Cells were seeded at 13,500–15,000 cells/insert in 0% FBS DMEM with added treatment and allowed to invade towards 10% FBS DMEM over 24 h at 37 °C and 5% CO_2_. Invasion of untreated cells towards 10% and 0% FBS was used as controls. Following incubation, non-invading cells were removed from the top chamber using a cotton swab and media was replaced in the bottom chamber with 1:10 PrestoBlue in 0% DMEM and incubated for 45 min with fluorescence measured at 544/590 nm. Number of invading cells was extrapolated from a standard curve comparing fluorescence intensity to cell seeding density (serially diluted from 20,000 to 234 cells/well). Finally, percentage invasion was calculated as the ratio of the number of invading cells to number of seeded cells.

#### Statistical analysis

Statistical data analysis and curve fitting was performed using GraphPad Prism (v8.4.3). Statistical significance was analyzed via one-way ANOVA with p < 0.05 considered significant. Inhibitory concentration values (IC_50_ and IC_25_ for afuresertib and taxifolin, respectively) were generated from sigmoidal dose–response curves with upper and lower 95% confidence intervals.

## Results and discussion

This study revealed three main outcomes: a widely applicable workflow based on metabolic modelling and network analysis (TISMAN), a list of potential drug targets for further investigation, and repurposing of drug compounds exerting inhibitory effects on GBM metabolic viability and invasion. Accessible high throughput sequencing of biopsies transformed the usage of RNA-sequencing in neuro-oncology practice^53^. Therefore, transcriptomics data was used to generate two contextualized models from a generic human GSM^25–27^, and to identify upregulated genes in cancerous cells^32^. Specifically, GBMs comprise pro-tumorigenic subpopulations of stem cells able to develop resistance to therapy^7–9,54^. While using FBA to obtain in silico gene essentiality is not a novel idea^23^, defining an appropriate objective function for normal and cancerous tissues to elucidate differences cannot be easily done^2,55^. Therefore, our analyses focused on cancer-specific models under an array of metabolic states and considering only reactions related to genes upregulated in GBM versus normal tissue. Conversely, recent metabolic modelling studies for GBM solely focused on the maximization of biomass yield, but generated c. 140 patient-derived GSMs^56^.

### Identification of genes of interest

**Figure 3:**
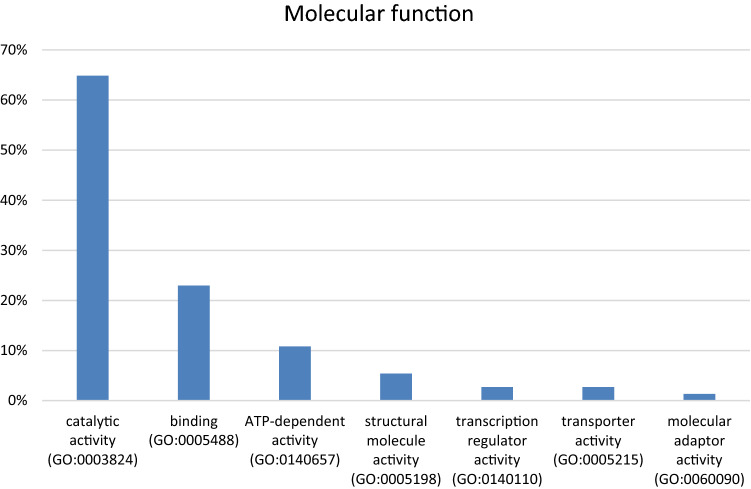
Functional classification of the 74 genes of interest. Using the PANTHER Classification System (17.0)^58^ showed that 65% of the 74 aforementioned genes have a catalytic function, which relates to the underlying metabolic modelling to TISMAN. A functional enrichment analysis performed in STRING v11^57^ identified oxidase and ligase activity in lipid metabolism to be particularly prevalent in this gene list.

A final list of 168 upregulated genes associated with essential reactions classed as extended chokepoints and/or topologically important, was generated. Specifically, 74 genes contained hits in the chemical-gene interaction databases (Fig. 3). The remaining 94/168 genes had no reported chemical–gene interactions, so they were deemed as potential drug targets yet to be explored (Dataset S1)^58^.

**Figure 4:**
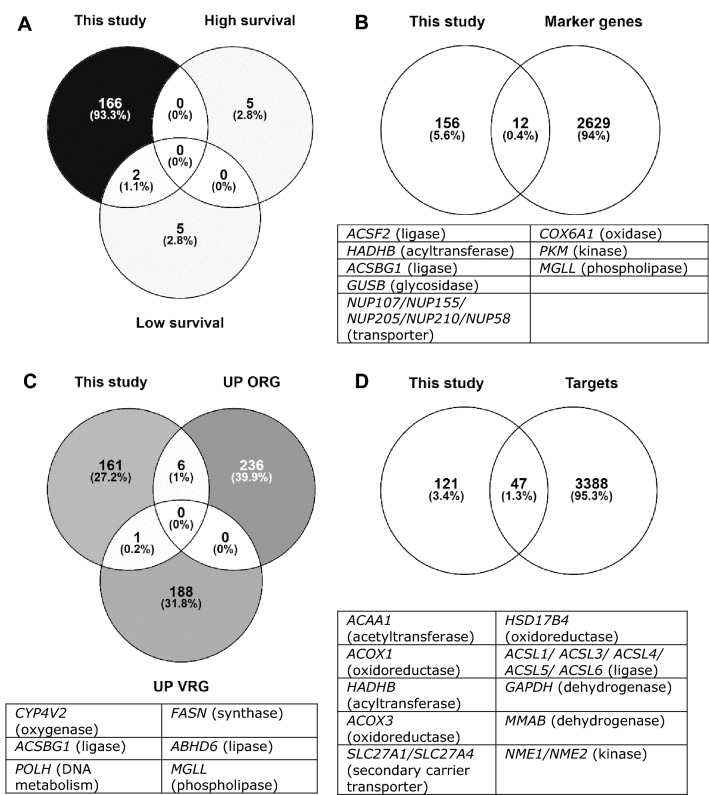
Comparison of genes of interest identified in this study against published works: (**A**) Larsson et al.^56^, which highlighted essential genes for models for high and low survival, (**B**) Richards et al.^9^, where the list of marker genes comprises the top 50 genes for each one of the 86 intra-GSC clusters, (**C**) Wang et al.^54^, where upregulated genes for outer RG-like and ventricular RG-like (UP ORG and UP VRG, respectively) were considered, and (**D**) Liu et al.^67^, where “targets” comprise both genes and miRNAs. Diagrams were made using Venny^68^. Panels B, C and D include tables informing about the protein type (most of them are enzymes).

Given the genetic heterogeneity which manifests within GBM^7–9,54^, predicted genes of interest were compared against modelling and experimental approaches applied to cancerous tissue or specific subpopulations (Fig. 4). In Larsson et al. (Fig. 4A), patient-derived GSMs for GBM were generated from transcriptomics data, and gene essentiality predicted by FBA^56^. Two of the genes pinpointed for the low survival models agreed with our results: *ACOT8* (acyl-CoA thioesterase 8) and *GPAT4* (glycerol-3-phosphate acyltransferase 4), both related to lipid metabolism. The former is targeted by three drugs with antineoplastic activity which decrease its expression: doxorubicin^52,59^, orlistat^60,61^, and paclitaxel^60,62^. *GPAT4* is the only gene of the *GPAT* family expressed in the brain, and it has recently been identified as a potential drug target for treating obesity-associated depression^63^.

A subpopulation of GSC can facilitate developing resistance to therapy, promoting disease relapse^7–9^. In Richards et al.^9^ certain marker genes for intra-GSC clusters were identified, some of which were also highlighted in this work as potential drug targets (Fig. 4B): *ACSF2* (acyl-CoA Synthetase Family Member 2), *MGLL* (monoglyceride lipase), and *PKM* (pyruvate kinase M1/2). Although *ACSF2* is functionally associated with fatty acid metabolism, *ACSF2* did not affect cell anchorage-dependent growth in U87 GBM cells^64^. *MGLL* is highly expressed in the brain and in aggressive cancers and its role in self-renewal and tumorigenic behavior has been demonstrated in murine models for GBM, where pharmacological inhibition of the translated protein showed promising results^65^. Finally, overexpression of pyruvate kinase M1/2 is a trait occurring in brain tumors but not in other cancers, where it is involved in metabolic and signaling functions that promote cell proliferation, migration and chemoresistance. Knocking down *PKM* alters central carbon metabolism and decreases the activation of oncogenes, thus making it a viable therapy target^66^.

Other genes of interest also identified by both Richards et al.^9^ and this study, currently lack (i) extensive evidence of therapeutic relevance to treating cancer and/or (ii) cognate ligands or compounds affecting their expression levels (or outdated chemical–gene interactions in databases): *ACSBG1* (acyl-CoA synthetase bubblegum family member 1), *COX6A1* (cytochrome c oxidase subunit 6A1), *GUSB* (glucuronidase beta), *HADHB* (acetyl-CoA acyltransferase), as well as multiple nucleoporins (*NUP58, NUP107, NUP155, NUP205* and *NUP201*). A recent study in renal cell carcinoma cells showed that *ACSBG1* induces ferroptosis, an iron-dependent regulated cell death proposed as a therapeutic approach for cancer treatment^69^. This mechanism plays a pro-tumorigenic effect in GBM by promoting necrosis, which is a sign of poor prognosis^70^. Interestingly, depleting *ACSL4* (acyl-CoA synthetase long chain family member 4), a gene highlighted by the workflow, also inhibited tumor necrosis^70^. *COX6A1*, has been reported as an anti-apoptotic gene in U373MG GBM cells, involved in mitochondrial energy metabolism^71^. *HADHB* is involved in fatty acid β-oxidation, and gene knockdown in a colon carcinoma cell line decreased cell proliferation and increased sensitivity to the monoclonal antibody cetuximab^72^. Regarding the aforementioned nucleoporins, even though there is a known link to cancer^73^, targeting membrane proteins remains a challenge^74^.

Radial glia (RG) cells are located in the ventricular zone during embryonic development and in the subventricular zone in the adult brain. In Wang et al., RG-like cells were found to play a role in the initiation or maintenance of adult GBM^54^. Specifically, two subpopulations of RG cells were identified: ventricular RG-like and outer RG-like, and upregulated RG genes are consistent with our results (Fig. 4C). Firstly, only one gene for ventricular RG-like was found in this study. *ACSL6* (acyl-CoA Synthetase Long Chain Family Member 6) is predominately expressed in the brain, where it participates in omega-3 fatty acid metabolism (crucial for correct brain function)^75^. Six genes were highlighted as outer RG-like: *ABHD6* (Abhydrolase Domain-Containing Protein 6), *CYP4V2* (cytochrome P450 family 4 subfamily V member 2), *FASN* (fatty acid synthase), *POLH* (DNA polymerase eta), *MGLL* and *ACSBG1*^54^. *ABHD6* is an endocannabinoid hydrolase with oncogenic functions, knockdown of which, reduces metastatic seeding in murine models^76,77^. *CYP4V2* metabolizes fatty acids; however, as mutants with an impaired activity are the cause of multiple diseases (e.g. Bietti’s crystalline dystrophy), this gene may not be a suitable drug target^78^. Regarding *FASN*, it is known to be highly expressed in GBM, where its inhibition in vitro and in vivo by means of a saffron-derived compound, induced cell death and impaired migration^79^. *POLH* is a DNA repair gene associated with correcting UV-induced damage, and its deficiency increases the risk of developing skin cancer^80^. Conversely, ovarian cancer stem cells survive cisplatin treatment due to an enhanced *POLH* expression, leading to tumor relapse^81^. Lastly, the remaining two genes identified as outer RG-like (*MGLL* and *ACSBG1*) are also marker genes for intra-GSC clusters, as discussed above.

Targeting transcription factors driving characteristic biological capabilities of cancer remains an attractive therapeutic approach^21^. 47/168 genes of interest had entries in RegNetwork (Fig. 4D), a database for regulatory information last updated in 2019^67^. Interestingly, 21 of these genes were related to the *MYC* family oncogene, which regulates a plethora of cellular functions and has been subjected to targeting studies at different levels^82^. Specifically, *MYC* maintains GSC^83^ and downregulating *MYC* promotes GBM autophagy^84^. Similarly, 13 genes are modulated by *PPARα*^67^, a transcription factor whose depletion inhibits tumor cell proliferation and induces senescence^85^. Overall, genes of interest identified by the workflow agreed with published attempts to find potential drug targets for GBM. Therefore, the validation of its predictive capabilities should foster more research into the aforementioned genes whose relevance to GBM has been poorly studied (e.g., *ACSBG1*). Improving the workflow by adding more biological information (such as epigenomics, proteomics or survival data) would allow further refining of potential drug targets to enable in vitro and in vivo efficacy characterizations.

### Selection of chemicals and in vitro testing

The goal of TISMAN is to facilitate drug repurposing projects by generating a prioritized list of potential drug targets to identify chemicals for testing in an efficient and timely manner. Multiple gene–chemical interactions databases were combined and used to identify relevant compounds, ranking them by the number of genes they interacted with (directly and/or indirectly, Fig. 2). Next, a literature search allowed selection based upon proven efficacy for other cancer types, but which have not yet been tested for GBM (Fig. 1).This approach reduced thousands of gene-chemical interactions to a ranked list of 30 compounds, allowing a detailed inspection of each one of them. Upon considering reported results, five chemicals were selected based on their potential effects on cell viability and invasion in GBM: afuresertib, pyrogallol, isorhamnetin, formononetin, and taxifolin (Table 1).

**Table 1:** Selected chemicals from the priority list for in vitro testing. Interactions for afuresertib were derived from *Comparative Toxicogenomics Database* (CTD) Oct 2020^46^, and interactions for all other compounds derived from *The Drug-Gene Interaction database* (DGIdb) Nov 2020, v4.2.0^45^. Gene relevance refers to the summation of the occurrence (extended choke point) and the topological importance (centrality score by PageRank). The stoichiometry of a reaction related to *ACSF2* can be found in Supplementary Table S2.

Position in priority list (relevance)	Drug/chemical	Gene name	Gene symbol	Gene relevance	Interaction(s) (predicted “–”)
4 (385)	Afuresertib	Acyl-CoA synthetase family member 2	*ACSF2*	119	Increases exp
		Acyl-CoA synthetase long chain family member 5	*ACSL5*	86	Increases exp
		Phenylalanyl-TRNA synthetase subunit beta	*FARSB*	9	Decreases exp
		Lipin 1	*LPIN1*	9	Increases exp
		Metabolism of cobalamin associated B	*MMAB*	26	Increases exp
		Patatin like phospholipase domain containing 2	*PNPLA2*	3	Increases exp
		DNA polymerase alpha 2, accessory subunit	*POLA2*	4	Decreases exp
		DNA polymerase delta 4, accessory subunit	*POLD4*	4	Increases exp
		Solute carrier family 27 member 1. Long-chain fatty acid transport protein 1	*SLC27A1*	119	Increases exp
		Solute carrier family 29 member 1. Equilibrative nucleoside transporter 1	*SLC29A1*	6	Decreases exp
8 (123)	Pyrogallol	Hydroxysteroid 17-beta dehydrogenase 10	*HSD17B10*	119	–
		DNA polymerase kappa	*POLK*	4	–
9 (119)	Formononetin	Hydroxysteroid 17-beta dehydrogenase 10	*HSD17B10*	119	–
13 (16)	3-*O*-methylquercetin (Isorhamnetin)	DNA polymerase beta	*POLB*	4	–
		DNA polymerase eta	*POLH*	4	–
		DNA polymerase iota	*POLI*	4	–
		DNA polymerase kappa	*POLK*	4	–
13 (16)	Taxifolin	DNA polymerase beta	*POLB*	4	–
		DNA polymerase eta	*POLH*	4	–
		DNA Polymerase iota	*POLI*	4	–
		DNA Polymerase kappa	*POLK*	4	–

Afuresertib is an orally bioavailable inhibitor of the serine/threonine protein kinase Akt pathway^86^ that can resensitize ovarian cancer to platinum-based chemotherapy^87^ and enhanced cisplatin treatment of mesothelioma cells^88^. The PI3K/AKT/mTOR pathway is related to metabolism, proliferation and migration, and is generally active in GBM^89^. Pyrogallol is a natural polyphenol with antitumor effects in hepatocellular carcinoma via the upregulation of miR-134^90^. This microRNA is deregulated in GBM (U87 cells), and overexpressing it inhibited cell proliferation and invasion^91^. Formononetin is an isoflavone affecting multiple signaling pathways with proven antitumorigenic properties in a rat glioma cell line^92,93^. 3-*O*-methylquercetin (isorhamnetin) is a flavonoid that inhibits cell proliferation and invasion, and induces apoptosis in triple-negative breast cancer cells^94^. Finally, taxifolin (also a flavonoid) down-regulates β-catenin, thus promoting mesenchymal to epithelial transition—the reverse process is the epithelial to mesenchymal transition, related to metastasis. Specifically, this compound inhibited cell proliferation and invasion in breast cancer cell lines^95^. Knockdown of PELP1, a β-catenin coactivator, proved both elements to be crucial for GBM progression in a murine model^96^. Even though interesting candidates were identified in the databases, some instances referred to predicted interactions with DNA polymerases rather than stating the aforementioned actual effects (Table 1). Well-curated and maintained databases would greatly benefit drug repurposing efforts, and researchers should consider updating the chemical-gene interaction entries based upon new data.

**Figure 5:**
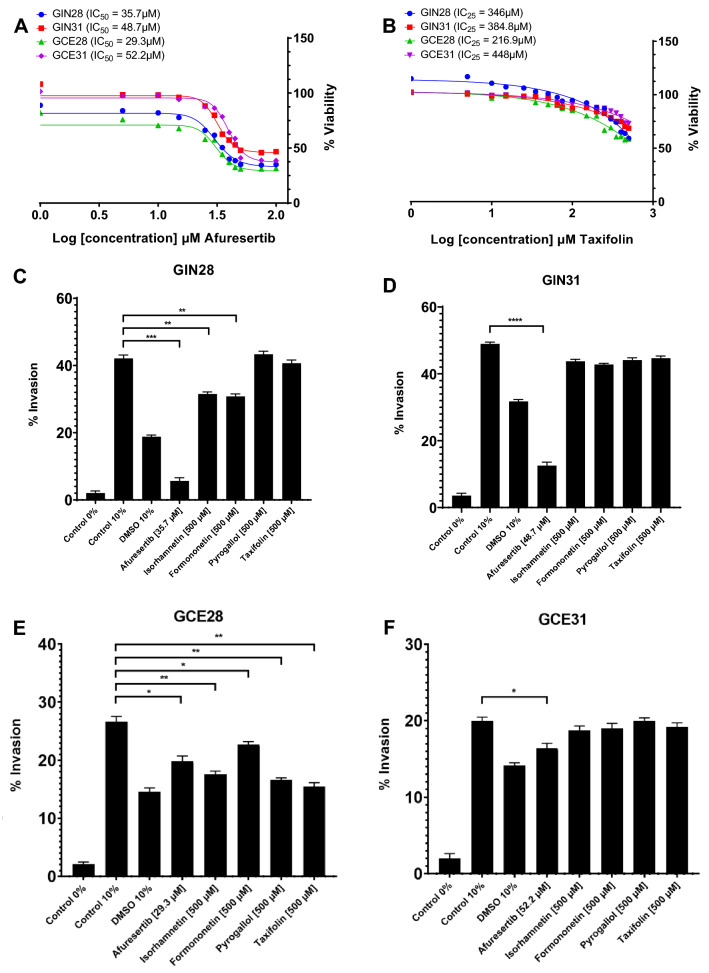
In vitro assessment of identified compounds for drug repurposing as validation of in silico workflow. (**A**,**B**) Metabolic viability was assessed for afuresertib (**A**) and taxifolin (**B**) in a 96-well 16-point dose response assay across four patient-derived GBM lines. PrestoBlue was used to assess metabolic viability after 72 h and data is presented as the mean percent viability compared to DMSO-treated controls (n = 6). Afuresertib was identified as the most potent drug inhibiting proliferation at IC_50_ values of 35.7 μM, 48.7 μM, 29.3 μM and 52.2 μM for the GIN28, GIN31, GCE28 and GCE31 cell lines, respectively (**A**). Taxifolin showed a modest but significant effect on cell sensitisation generating IC_25_ values of 346 μM, 384.8 μM, 216.9 μM, 448 μM on GIN28, GIN31, GCE28 and GCE31 cell lines, respectively (**B**). (**C**–**F**) Effects on invasion in GBM invasive margin (GIN28, GIN31) and GBM Contrast-Enhanced core (GCE28, GCE31) cell lines following treatment with four compounds at 500 μM and afuresertib at IC50 concentration. Invasion towards 0% FBS (Control 0%) on untreated cells and DMSO treated (DMSO 10%) controls were also included. Data is presented as mean percent invasion ± SEM compared to number of seeded cells after 24 h incubation. Significance was tested by one-way ANOVA (*p < 0.05, **p < 0.01, ***p < 0.001, **** p < 0.0001) comparing each data set to control 10% (invasion towards 10% FBS; n = 3).

Two in vitro tests were performed to assess the effects of the selected chemicals on metabolic viability and invasiveness of four patient-derived GBM cell lines, as a means to validate this innovative in silico pipeline. Afuresertib was identified as the most potent drug inhibiting proliferation (Fig. 5A), while taxifolin showed a modest but significant effect on cell sensitization (Fig. 5B). No significant effect on proliferation was observed after treatment with isorhamnetin, formononetin and pyrogallol at 72 h. Regarding the invasion assays, afuresertib was the only compound to significantly inhibit invasion at IC_50_, impairing invasion by 86.7% (p < 0.001) and 74.5% (p < 0.0001) in GIN28 and GIN31 GBM invasive margin cells, respectively, and by 25.6% (p = 0.017) and 18% (p = 0.028) in corresponding GCE28 and GCE31 counterpart cell lines derived from the central tumor core. At 500 μM, isorhamnetin significantly reduced invasion by 25.2% (p = 0.0036) in GIN28 and 34.2% (p = 0.0011) in GCE28. A significant reduction in invading cells was also observed in GIN28 and GCE28 cell lines upon treatment with formononetin at 500 μM, impairing invasion by 26.8% (p = 0.0042) and 14.7% (p = 0.0193), respectively. While taxifolin demonstrated an antiproliferative effect across all cell lines, a significant effect on invasion was only observed in GCE28 (p = 0.0034) when used at 500 μM, reducing invasion to 15.5% (Fig. 5C–F). Therefore, afuresertib should be considered for in vitro studies to understand the underlying mechanisms to different responses, as well to assess the effect on GSCs, potentially leading to further in vivo studies to characterize its pre-clinical suitability. Collectively, in vitro assessment identifies 2/5 tested compounds as impairing metabolic viability, and 3/5 tested compounds as impairing invasion. Considering both intra- and inter-tumor heterogeneity which manifests in GBM, and thereby the likely biological variation in TCGA transcriptomics data compared to the patient-derived GBM lines used in this study, validation of 40% and 60% of repurposed compounds for anti-proliferative and anti-invasion effects, represents an effective hit rate and validates TISMAN as an in silico workflow to expedite compound prioritization at a drug discovery phase. Furthermore, identified genes lacking targeting molecules should be considered in structural studies to enable and exploit molecular docking efforts.

## Supplementary Information

Supplementary Information.

Supplementary Tables.
