# Supplementary material for: Metabolic modeling-based drug repurposing in Glioblastoma
Source: Sci Rep. 2022 Jul 1;12:11189. doi: 10.1038/s41598-022-14721-w (PMC9249780; doi:10.1038/s41598-022-14721-w)
Supplement: Supplementary file 2 — Supplementary Tables. [file 41598_2022_14721_MOESM2_ESM.pdf]

Table S1. Single tandem repeat genotyping of GIN-28, GCE-28, GIN-31 and GCE-31 patient-derived primary lines isolated from the GBM 5ALA invasive edge (GIN) and contrast enhanced core (GCE). DNA was isolated from each sample and genetic characteristics determined by PCR-single-locus-technology, utilizing 21 independent PCR-systems. The GIN-28 line compared across two passages (p11 and p33), showed identical STR genotypes, with 20/21 DNA systems matching the STR genotype from primary invasive margin GBM tissue (T28.3) from which the cells were derived. GIN-31 p11 cells fully retain the genotype from primary invasive margin GBM tissue (T31.5) from which the cells were derived; GIN-31 p33 retains a matched STR genotype in 13/21 loci, with amplifications acquired in culture likely a result of the 22 passages between this sample and early passage. The GCE-28 line compared across two passages (p16 and p19), showed identical STR genotypes, with 20/21 DNA systems matching the STR genotype from primary invasive margin GBM tissue (T28.3) from which the cells were derived. The GCE-31 line compared across two passages (p7 and p16), showed identical STR genotypes, and fully retains the genotype from primary invasive margin GBM tissue (T31.5) from which the cells were derived. Collectively these results confirm that the cell lines utilized in this study have not been cross-contaminated with any other cell line and the primary lines derived from the GBM invasive margin and core regions, broadly retain the genotype of the tumor. Materials & Methods - Cell line authentication of the, GIN-28, GCE-28, GIN-31 and GCE-31 patient-derived primary lines were determined by PCR-single-locus-technology, utilizing 21 independent PCR-systems (Eurofins, Germany).

**DNA-System**	DNA-criteria **T28.2** CL181019_025	DNA-criteria **GIN-28 p11** CL18019_021	DNA-criteria **GIN-28 p33** CL18019_022	DNA-criteria **GCE-28 p16** CL18019_023	DNA-criteria **GIN-28 p19** CL18019_024	DNA-criteria **T31.5** CL18019_030	DNA-criteria **GIN-31 p11** CL18019_026	DNA-criteria **GIN-31 p31** CL18019_027	DNA-criteria **GCE-31 p7** CL18019_028	DNA-criteria **GCE-31 p16** CL18019_029
**AM**	X,Y	X,Y	X,Y	X,Y	X,Y	X,X	X,X	X,X	X,X	X,X
**D3S1358**	14,17	14,17	14,17	14,17	14,17	16,18	16,18	16,18	16,18	16,18
**D1S1656**	12,16	12,16	12,16	12,16	12,16	12,15	12,15	12,15	12,15	12,15
**D6S1043**	11,13	11,13	11,13	11,13	11,13	12,18	12,18	12,18	12,18	12,18
**D13S317**	11,12	11,12	11,12	11,12	11,12	13,13	11,13	11,13	11,13	11,13
**Penta E**	7,17	7,17	7,17	7,17	7,17	7,12	7,12	7,12	7,12	7,12
**D16S539**	9,12	9,12	9,12	9,12	9,12	10,11	10,11	10,11	10,11	10,11
**D18S51**	14,17	14,17	14,17	14,17	14,17	17,18	17,18	16,17,18	17,18	17,18
**D2S1338**	19,23	19,23	19,23	19,23	19,23	19,25	19,25	11,19,25	19,25	19,25
**CSF1PO**	11,13	11,13	11,13	11,13	11,13	11,12	11,12	11,12	11,12	11,12
**Penta D**	12,13,14	12,14	12,14	12,14	12,14	11,12	11,12	11,12	11,12	11,12
**TH01**	6,9,3	6,9,3	6,9,3	6,9,3	6,9,3	6,9	6,9	6,9	6,9	6,9
**vWA**	14,15	14,15	14,15	14,15	14,15	14,18	14,18	14,15,18,21	14,18	14,18
**D21S11**	27,33	27,33	27,33	27,33	27,33	29,29	29,29	29,29	29,29	29,29
**D7S820**	8,10	8,10	8,10	8,10	8,10	7,10	7,10	7,10	7,10	7,10
**D5S818**	11,12	11,12	11,12	11,12	11,12	11,12	11,12	11.1,12	11,12	11,12
**TPOX**	8,11	8,11	8,11	8,11	8,11	9,9	9,9	9,9	9,9	9,9
**D8S1179**	10,14	10,14	10,14	10,14	10,14	11,13	11,13	11,13,14.1	11,13	11,13
**D12S391**	20,21	20,21	20,21	20,21	20,21	23,23	17,23	16,17,22.3,23	17,23	17,23
**D19S433**	13,14	14,14	14,14	14,14	14,14	12,?	12,15	12,13.1,15	12,15	12,15
**FGA**	23,25	23,25	23,25	23,25	23,25	22,26	22,26	19.3,22,26	22,26	22,26

Table S2. Example of a reaction of interest: 'HMR_6436'. This reaction along with 'HMR_6453' are related to *ACSF2* (ENSG00000167107), a gene indirectly targeted by afuresertib. More information about the metabolites can be found at https://metabolicatlas.org.

Metabolite symbol	Metabolite name	Comment
**m01096m[m]**	5-carboxy-alpha-chromanol [Mitochondria]	Exclusively produced by 'HMR_6453'. Exclusively consumed by **'HMR_6436'**.
**m01371m[m]**	ATP [Mitochondria]	-
**m01597m[m]**	CoA [Mitochondria]	-
**m02040m[m]**	H2O [Mitochondria]	-
**m02552m[m]**	NAD+ [Mitochondria]	-
**m02630m[m]**	O2 [Mitochondria]	-
	→	
**m00766m[m]**	3-carboxy-alpha-chromanol [Mitochondria]	Exclusively produced by **'HMR_6436'**. Exclusively consumed by 'HMR_6438' (transport to cytosol).
**m01334m[m]**	AMP [Mitochondria]	-
**m02039m[m]**	H+ [Mitochondria]	-
**m02041m[m]**	H2O2 [Mitochondria]	-
**m02553m[m]**	NADH [Mitochondria]	-
**m02759m[m]**	PPi [Mitochondria]	-
**m02774m[m]**	propanoyl-CoA [Mitochondria]	-
